# For Women by Women

**Published:** 1889-05-11

**Authors:** 


### For Women by Women.

The committee of the New Hospital for Women are to be congratulated on the excellent arrangements made for the ceremony of laying the foundation-stone, which took place last Tuesday. The large marquee was well ventilated, tastefully decorated, and, when filled with a well-dressed crowd, made really a pretty picture. The crowds who gathered along the Euston-road to welcome the Prince and Princess of Wales were not kept waiting, for punctually at 4.30 the Royal carriages came in sight, and the band of the Artists Volunteers, who were in attendance, struck up the National Anthem. The Princess looked very well, and quite as young as her two daughters, who accompanied her. She was dressed in a tight-fitting black velvet, with a white waistcoat, and the Princesses Victoria and Maud were in grey. As they entered the marquee, the Prince and Princess were received by the committee, who all wore badges of forget-me-nots. Lady Dufferin and some other personal friends shook hands with their Royal Highnesses, and little Miss Sylvia Stanley presented the Princess with a bouquet of roses. Mrs. Garrett Anderson commenced the proceedings by reading an address, during which she said: "The committee trust that in its permanent form happily inaugurated to-day by your Royal Highness, the New Hospital for Women will for generations to come help women to carry on worthily a noble and difficult work, both in this country and in India, and that it will also serve as a memorial of the social and intellectual advance made by women in the memorable reign of Her Most Gracious Majesty Queen Victoria."

The Archbishop of Canterbury offered up a prayer that the ministry of women to their fellow sisters might be hallowed by God's blessing; and then the grand voice of Madame Antoinette Sterling rang through the tent—"He leadeth us, even though we walk through the valley of the shadow of death."

After the architect and builder had been presented, the Princess proceeded to lay the stone, spreading the mortar skilfully, and guiding the descending stone, smiling the while, as though the accustomed ceremony still had its comic side to her mind. A pile of purses was then placed on the stone by friends who had collected in aid of the charity, and the Rev. J. Llewelyn Davies, who is chairman of the committee, thanked the Princess for her presence, acknowledging that her support had helped women to overcome the barriers which beset their entrance to the medical profession. The Prince of Wales, in a short speech, replied for the Princess, saying she was quite aware of the difficulties and prejudices which medical women had had to overcome, and that she sincerely hoped the hospital would prove a blessing to her sex. The speech was received with cheers. Next came a trio, "O Lift Thine Eyes," sung by Madame Antoinette Sterling, Miss Eleanor Rees, and Miss Liza

Lehmann. The Archbishop then pronounced the blessing. The formal proceedings being over, the Princess shook hands with Madame Sterling and thanked her for her singing, and then retired for a cup of tea. Several of the committee and medical staff of the hospital were presented to their Royal Highnesses by special request.

There were many well-known faces in the audience, amongst which we recognised Sir Spencer Wells, Mr. Plaskitt, Miss Bonham-Carter, Mrs. Tait, and the energetic secretary, Miss M. Bagster. The site and building of the new hospital will cost £20,000, of which about £12,000 has been so far collected, Mrs. Westlake, Mr. Thomasson, Mr. and Mrs. Garrett Anderson, Mrs. Charles Holland, and Lady Harriet Wentworth having each subscribed £1,000 towards the building fund. Miss Florence Nightingale and Sir Douglas Galton have advised as to the plans for the new building, and the hospital should be a model one. Certainly it has secured a good start in receiving the support of the Princess of Wales.

